# Multi-Sensor Data Fusion Identification for Shearer Cutting Conditions Based on Parallel Quasi-Newton Neural Networks and the Dempster-Shafer Theory

**DOI:** 10.3390/s151128772

**Published:** 2015-11-13

**Authors:** Lei Si, Zhongbin Wang, Xinhua Liu, Chao Tan, Jing Xu, Kehong Zheng

**Affiliations:** 1 School of Mechatronic Engineering, China University of Mining & Technology, Xuzhou 221116, China; E-Mails: lei.si@cumt.edu.cn (L.S.); l_xinhua_2006@126.com (X.L.); tccadcumt@126.com (C.T.); xujingcumt@126.com (J.X.); zkhcumt@gmail.com (K.Z.); 2 School of Information and Electrical Engineering, China University of Mining & Technology, Xuzhou 221116, China

**Keywords:** shearer, cutting condition identification, parallel quasi-Newton algorithm, neural network, Dempster-Shafer theory, feature extraction

## Abstract

In order to efficiently and accurately identify the cutting condition of a shearer, this paper proposed an intelligent multi-sensor data fusion identification method using the parallel quasi-Newton neural network (PQN-NN) and the Dempster-Shafer (DS) theory. The vibration acceleration signals and current signal of six cutting conditions were collected from a self-designed experimental system and some special state features were extracted from the intrinsic mode functions (IMFs) based on the ensemble empirical mode decomposition (EEMD). In the experiment, three classifiers were trained and tested by the selected features of the measured data, and the DS theory was used to combine the identification results of three single classifiers. Furthermore, some comparisons with other methods were carried out. The experimental results indicate that the proposed method performs with higher detection accuracy and credibility than the competing algorithms. Finally, an industrial application example in the fully mechanized coal mining face was demonstrated to specify the effect of the proposed system.

## 1. Introduction

In a fully mechanized working face, the shearer is one of the most important pieces of coal mining equipment and monitoring its cutting condition has played an indispensable important segment for the automatic control of shearer. However, due to the poor mining environment and complex component structure of a shearer, the operator cannot identify the shearer cutting conditions timely and accurately only with the help of visualization. Under this circumstance, the shearer drum may cut the rock, which will cause harm to the machine and lead to poor coal quality and low mining efficiency. Another concern is that many casualties occur in collieries. Therefore, it is necessary to efficiently and accurately identify shearer cutting conditions, which is becoming a challenging and significant research subject [1].

Over the past few decades, some scholars have focused on the coal-rock interface recognition to roughly estimate the cutting state of shearer. In [2], Yu *et al.* used the sonic wave reflection method to identify the coal-rock interface. In [3], the image processing technique for visible light and infrared images was applied to the recognition of coal-rock interfaces. In [4], the vibration signals of a hydraulic support beam were used to extract its features by the use of wavelet packet energy spectrum and the coal-rock interface was identified. In [5], the method based on natural γ–rays was utilized to identify the coal-rock interface. Sahoo *et al.* used the opto-tactile sensor to recognize the rock surfaces in underground coal mining [6]. In [7], radar technology was used to identify the coal-rock interface and obtain the cutting patterns of the shearer. However, coal-rock interface recognition technology requires too harsh geological conditions of the coal seam, and the recognition precision cannot help the shearer achieve automatic control.

According to some research focuses in the literature, intensive study has been done in the fault diagnosis of traditional equipment. Sensors are used extensively in pattern recognition and fault diagnosis systems because they can provide the inner information of the machine. Using vibrations to collect state information has become effective [8]. For this reason, vibration-based analysis is becoming the most commonly used method and is also proving efficient in various real applications. For a shearer, the rocker arm is the critical component and the vibration of rocker arm can comprehensively reflect the cutting condition of the shearer, which can be diagnosed correctly by appropriate measurement and description of sensors. Therefore, data analysis methods of the measured signals are essential.

In recent years, the commonly used data analysis methods for vibration signals have been wavelet transform (WT), Fourier transform (FT), Hilbert-Huang transform (HHT), empirical mode decomposition (EMD), and so on. In [9], a method based on wavelet transform was proposed to analyze the vibration response of discrete piecewise linear oscillators. In [10], the authors made an attempt to identify the vibration sources, analyze the law of vibration propagation, and establish the relationship between the vibration sources and ground vibration using the Time-Wavelet Power Spectrum and the Cross Wavelet Transform techniques. In [11], the chaotic vibrations of flexible plates of infinite length were studied and analyzed by the use of fast Fourier transforms and wavelets. In [12], a Hilbert-Huang transform (HHT) algorithm was presented for flywheel vibration analysis to lay the foundation for the detection and diagnosis in a reactor main coolant pump. In [13], the authors employed nonlinear rotor dynamics with the vibration signal processing scheme based on the Empirical Mode Decomposition (EMD) in order to understand the vibration mechanism.

Nevertheless, when separately depending on data, an analysis is difficult to directly identify the working status or fault type of a machine. The trend in recent years has been to automate the analysis of the measured signals by incorporating the data analysis methods with machine learning algorithms, such as neural networks (NNs) and the support vector machine (SVM) [14,15,16]. NNs have gained popularity over other techniques because they are efficient in discovering similarities among large bodies of data. It has the ability to simulate human thinking, and owns powerful function and incomparable superiority in terms of establishing nonlinear and experiential knowledge simulation models. The common training method for NNs is the standard back-propagation training algorithm, which is known to have some limitations of local optimal solution, a low convergence rate, obvious “overfitting” and especially poor generalization when the number of fault samples is limited [17,18]. Watrous R.L. [19] tested the application of a quasi-Newton method proposed by Boyden, Fletcher, Goldfarb and Shanno (abbreviated as BFGS method) to neural network training. It was shown that the BFGS method converges much faster than the standard back-propagation method, which uses the gradient method. 

However, the quasi-Newton method has the obvious drawback that it consumes a lot of time and memory to store the Hessian matrix, which leads to the limitation in applications of complex problems. Considering the superiority of parallelism mechanism in processing speed, many methods based on parallelism mechanism have arisen to improve the convergence speed of neural network [20,21,22]. In this paper, computing parallelism is coupled with the quasi-Newton algorithm to generate the parallel quasi-Newton (PQN) algorithm, which is used in the training process of neural networks.

However, the signal data collected from a single sensor may be invalid or inaccurate. A single sensor has limited capabilities for resolving ambiguities and has the ability to provide consistent descriptions of the measurement, which makes the classification results of NNs spurious and incorrect. Therefore, multi-sensor data fusion arises at the historic moment, which can potentially improve the detection capabilities and probability that any damage is detected. There are many multi-sensor data fusion methods being applied in fault diagnosis and pattern recognition. In [23], the authors proposed an intelligent multi-sensor data fusion method using the relevance vector machine based on an ant colony optimization algorithm for gearbox fault detection. In [24], a method was presented that used multi-sensor data technology and the *k*-Nearest Neighbor algorithm to diagnose the fault pattern of rolling element bearings. In [25], the authors used the federated Kalman filter to fuse the sensor signals for high-speed trains in a high accuracy navigation system. After many years of development of information fusion technology, Dempster-Shafer (DS) theory is now commonly known and used. In [26], a novel and easily implemented method was presented to fuse the multisource data in wireless sensor networks through the DS evidence theory. In [27], a novel information fusion approach using the DS evidence theory and neural networks was proposed to forecast the distribution of coal seam terrain. In [28], an intelligent detection method was proposed by integration of multi-sensory data fusion and classifier ensemble to detect the location and extent of the damage based on posteriori probability support vector machines and the DS evidence theory. In [29], a multi sensor fusion methodology was proposed to identify indoor activity based on DS theory framework with an incremental conflict resolution strategy. According to the literature, DS theory does not need prior knowledge of the probability distribution, and it is able to assign probability values to sets of possibilities rather than to single events only.

Bearing the above observation in mind, we provide a cutting condition identification scheme for the shearer based on the vibration signals of the rocker arm and the current signal of the cutting motor. The PQN-NN algorithm and DS theory is used to improve the performance and accuracy of the condition diagnosis system. Firstly, feature extraction is conducted by signal processing techniques. Secondly, the extracted data are used for inputs of the neural network to obtain some classifiers and the outputs are assessed quantitatively. Lastly, estimated quantities from different classifiers are combined by DS theory to enhance the identification accuracy.

The rest of this paper is organized as follows. In Section 2, we briefly present the basic theory of the advanced neural network and DS theory. Section 3 describes the main key techniques of the proposed method and provides some experimental analysis. Section 4 presents the application effect of the proposed method in the coal mining face. Our conclusions and future works are summarized in Section 5.

## 2. Theoretical Background 

### 2.1. Parallel Quasi-Newton Neural Network (PQN-NN) 

A feedforward network model with multi-input and multi-output is taken as an example to represent the basic principle. The input and output vectors are set as *X* = (*x*_1_, *x*_2_, …, *x_n_*) and *Z* = (*z*_1_, *z*_2_, …, *z_m_*), and the output of hidden layer is *H* = (*h*_1_, *h*_2_, …, *h_s_*). The activation functions of the hidden layer and output layer can be chosen as sigmoid function. The connection weight of network is defined as *w* = [*w*^1^, *w*^2^], where *w*^1^ is the connection weight between input and hidden layers and *w*^2^ is the connection weight between hidden and output layers. The desired output is *Z_d_* and the number of training samples is *P*. The error between desired output and network output is selected as $E=\frac{1}{2}\sum_{i=1}^{P}{\left\|Z_{i}-Z_{di}\right\|}^{2}$. The weights of network should be updated to minimize error *E*.

According to the principle of the quasi-Newton algorithm (QN), the updating formula of weights *w_k_* can be calculated as follows:

(1) $$w_{k+1}=w_{k}+\lambda_{k}d_{k}$$

where *k* is the number of iterations, *d_k_* = −*H_k_*·*g_k_* is the search direction, *λ_k_* is the step-size of the iteration *k*, $g_{k}=\nabla E(w_{k})$, $H_{k}=\nabla^{2}E(w_{k})$ is the current inverse Hessian matrix approximation [30]. In the QN method, the selection of *H_k_* directly affects the performance of the algorithm. In order to obtain better optimization results, some scholars put forward different methods to determine *H_k_*. But these strategies would increase the calculation of the algorithm and degrade the efficiency. In this paper, a parallel quasi-Newton optimization algorithm (PQN) was provided to train the neural network. The PQN algorithms use the following updating formula with three parameters:

(2) $$H_{k+1}(\phi_{k},\theta_{k},\gamma_{k})=[H_{k}-\frac{H_{k}y_{k}y_{k}^{T}H_{k}}{y_{k}^{T}H_{k}y_{k}}+\phi_{k}(y_{k}^{T}H_{k}y_{k})v_{k}v_{k}^{T}]\theta_{k}+\frac{s_{k}s_{k}^{T}}{\gamma_{k}s_{k}^{T}y_{k}}$$

where $y_{k}=\nabla E(w_{k+1})-\nabla E(w_{k+1}),s_{k}=w_{k+1}-w_{k},v_{k}=\frac{s_{k}}{s_{k}^{T}y_{k}}-\frac{H_{k}y_{k}}{y_{k}^{T}H_{k}y_{k}}$. The three parameters can be defined by:

(3) $$\phi_{k}=\frac{s_{k}^{T}y_{k}}{y_{k}^{T}(s_{k}-H_{k}y_{k})},\theta_{k}=\frac{s_{k}^{T}y_{k}}{y_{k}^{T}H_{k}y_{k}},\gamma_{k}=\frac{6}{s_{k}^{T}y}[f(w_{k})-f(w_{k+1})+s_{k}^{T}g_{k+1}]-2$$

The learning process of PQN neural network can be shown as follows:

**Step 1**: Initialize variables. The variables mainly include the initial random values of the weights (*w*_0_), initial approximate inverse Hessian, named the identity matrix *I*, convergence condition (*ε*), maximum iterations (*K*_max_) and *k* is set as 0 initially.

**Step 2**: Compute the parallel search directions. In this paper, two search directions are chosen as follows:

(4) $$d_{k1}=-H_{k}(1,{\bar{\theta }}_{k},\gamma_{k})g_{k},d_{k2}=-H_{k}(\phi_{k},{\bar{\theta }}_{k},1)g_{k}$$

where ${\bar{\theta }}_{k}$ is the scaling parameter and can be adjusted as follows:

(5) $${\bar{\theta }}_{k}=\left\{ \begin{array}{l} \varepsilon_{1},\;if\;\theta_{k}\leq \varepsilon_{1}\\ \theta_{k},\;if\;\varepsilon_{1}<\theta_{k}<\varepsilon_{2}\\ \varepsilon_{2}\;if\;\theta_{k}\geq \varepsilon_{1} \end{array}\right.$$

where the arguments *ε*_1_, *ε*_2_ satisfy 0 < *ε*_1_ < *ε*_2_ ≤ 1. In our experiment, we set *ε*_1_ = 0.5, *ε*_2_ = 1 [31].

**Step 3**: Perform the parallel line searches. Along each search direction, inexact line searches are performed to determine the step-size *λ_jk_* in parallel according to the following Wolfe conditions proposed by Hanno and Phua:

(6) $$\left\{ \begin{array}{l} E(w_{k}+\lambda_{jk}d_{kj})\leq E(w_{k})+\rho_{1}\lambda_{jk}d_{kj}\\ g(w_{k}+\lambda_{jk}d_{kj})d_{kj}\geq \rho_{2}g_{k}^{T}d_{kj} \end{array}\right.\;j=1,2$$

where $\rho_{1}$ and $\rho_{2}$ are the regulatory factors and $0<\rho_{1}<0.5,\rho_{1}<\rho_{2}<1$.

The process terminates until two step-sizes are found along all the search directions.

**Step 4**: Choose the minimum point. Let $d_{k}^{*}$ denote the direction that attains the minimum function value and $\lambda_{k}^{*}$ be the step-size corresponding to $d_{k}^{*}$. The only $d_{k}^{*}$ and $\lambda_{k}^{*}$ can be determined to update the weights $w_{k+1}=w_{k}+\lambda_{k}^{*}d_{k}^{*}$ through the following formula:

(7) $$E(w_{k}+\lambda_{k}^{*}d_{k}^{*})=\underset{j=1,2}{\min}(w_{k}+\lambda_{jk}d_{kj})$$

**Step 5**: Test for convergence. If the convergence criterion satisfies the condition $\left\|g_{k+1}\right\|\leq \varepsilon$, then stop; otherwise, compute *H*_*k*+1_ according to Equation (2).

**Step 6**: Repeat the process: Set *k* = *k* + 1 and repeat the process from Step 2.

### 2.2. Dempster—Shafer Theory

The Dempster-Shafer theory, known as evidence theory, was initially proposed by Dempster [32,33] and Shafer [34], was elaborated by Smets [35,36], and then was further developed by Denoeux [37,38]. The basic concepts and mechanisms of the Dempster-Shafer (DS) theory are introduced in this subsection.

In DS theory, a finite non-empty set *Θ* is assumed as a set of hypotheses, which contains *N* exclusive elements and *Θ*= {*A*_1_, *A*_2_, …, *A_N_*} is called the frame of discernment. The following function should be firstly defined: $m:2^{\Theta }\to [0,1],m(\emptyset )=0,\sum_{A\subseteq \Theta }m(A)=1$, where *m*(*A*) denotes the basis belief assignment (BBA). If we provide a piece of evidence, every possible hypothesis or their combination should be assigned the belief level in the range of [0, 1]. The empty set should be assigned the belief level of zero and the sum of all BBAs should be equal to 1.

The BBA *m*(*A*) is used to describe the belief level that the evidence supports *A*. For each subset A⊆Θ, *m*(*A*) is bigger than zero and can be called the focal element of *m*. Two concepts should be defined as follows:

(8) $$Bel(A)=\sum_{B\subseteq A}m(B),\;Pl(A)=\sum_{A\cap B\neq \emptyset }m(B)$$

where *Bel*(*A*) denotes the belief function and *Pl*(*A*) denotes the plausibility function.

Belief function *Bel*(*A*) provides the support for *A* that hypothesis *A* is true and *Bel*(*A*) is also interpreted as the lower limit function. Plausibility function *Pl*(*A*) represents the support for *A* that hypothesis *A* is not false and can be known as upper limit function.

Through the description of the basis belief assignment, the information of different sources can be combined by a fusion rule proposed by Dempster. We assume that *m*_1_ and *m*_2_ are two BBAs induced by the evidence, which must be independent. Then the Dempster’s rule is used to combine the two massed to generate a new mass function, which can be calculated as follows:

(9) $$\begin{array}{l} (m_{1}⊕m_{2})(A)=\frac{1}{1-K}\sum_{B\cap C=A}m_{1}(B)m_{2}(C)\\ K=\sum_{B\cap C=\emptyset }m_{1}(B)m_{2}(C) \end{array}$$

In this Dempster’s rule, the value of *K* reflects the degree of conflict between *m*_1_ and *m*_2_ induced by evidence. The coefficient 1/(1 − *K*) is referred to as the normalization factor and its role is to avoid the non-zero probability to be assigned to an empty set in the synthesis process. With the increase of *K*, the conflicts will become more and more obvious and the combination results may be not consistent with the actual situation.

## 3. Experimental Analysis

### 3.1. System Structure

**Figure 1:**
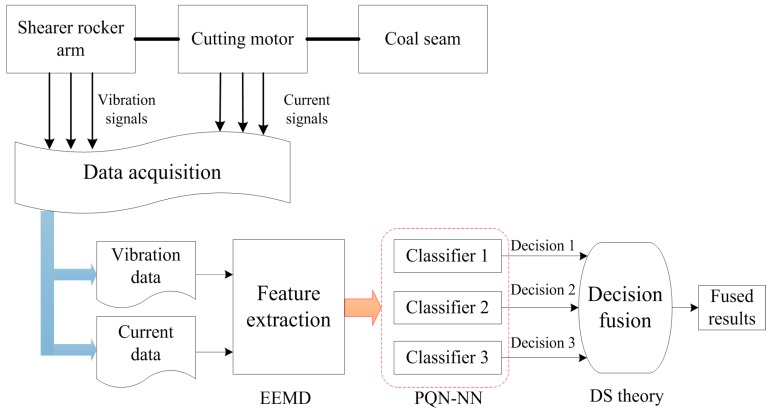
Flowchart of the proposed condition identification system for shearer drum.

The vibration and current signals are used to classify the cutting conditions of the shearer drum since the signals can describe its dynamic characteristics. In order to classify the cutting mode of the shearer drum, the following three processes are required: data acquisition, feature extraction, and pattern recognition. The proposed condition classification system for the shearer drum is shown in Figure 1. The system mainly consists of three critical steps: data acquisition, feature extraction and decision fusion. Firstly, the vibration signals and current signal are obtained by the accelerometers and Hall current sensor from the experimental rig for a shearer cutting coal. Then the features of the data are extracted based on the ensemble empirical mode decomposition algorithm. Feature extraction algorithms can make data quantitative from the view of statistics. We should note that the signals processing and classifiers establishment are mainly accomplished in the PC. Finally, the PQN-NN and DS theory are used to classify the cutting conditions of machinery.

### 3.2. Data Acquisition

**Figure 2:**
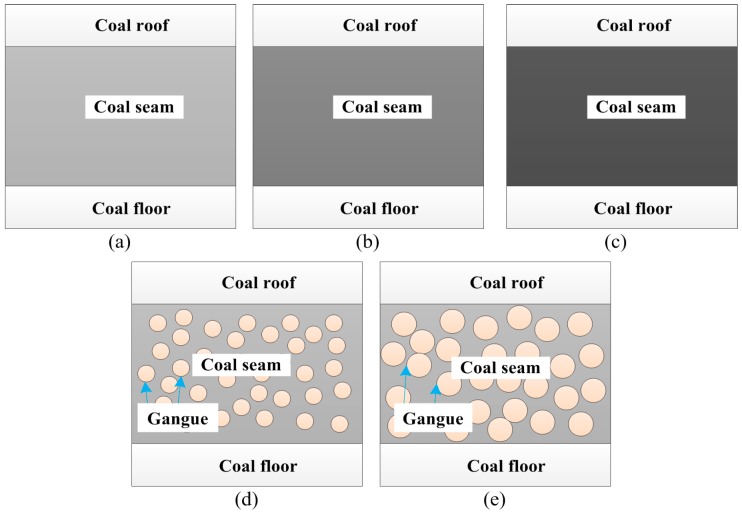
Different geological conditions of coal seam: (**a**) *f* = 2; (**b**) *f* = 3; (**c**) *f* = 4; (**d**) *f* = 5; (**e**) *f* = 6.

**Figure 3:**
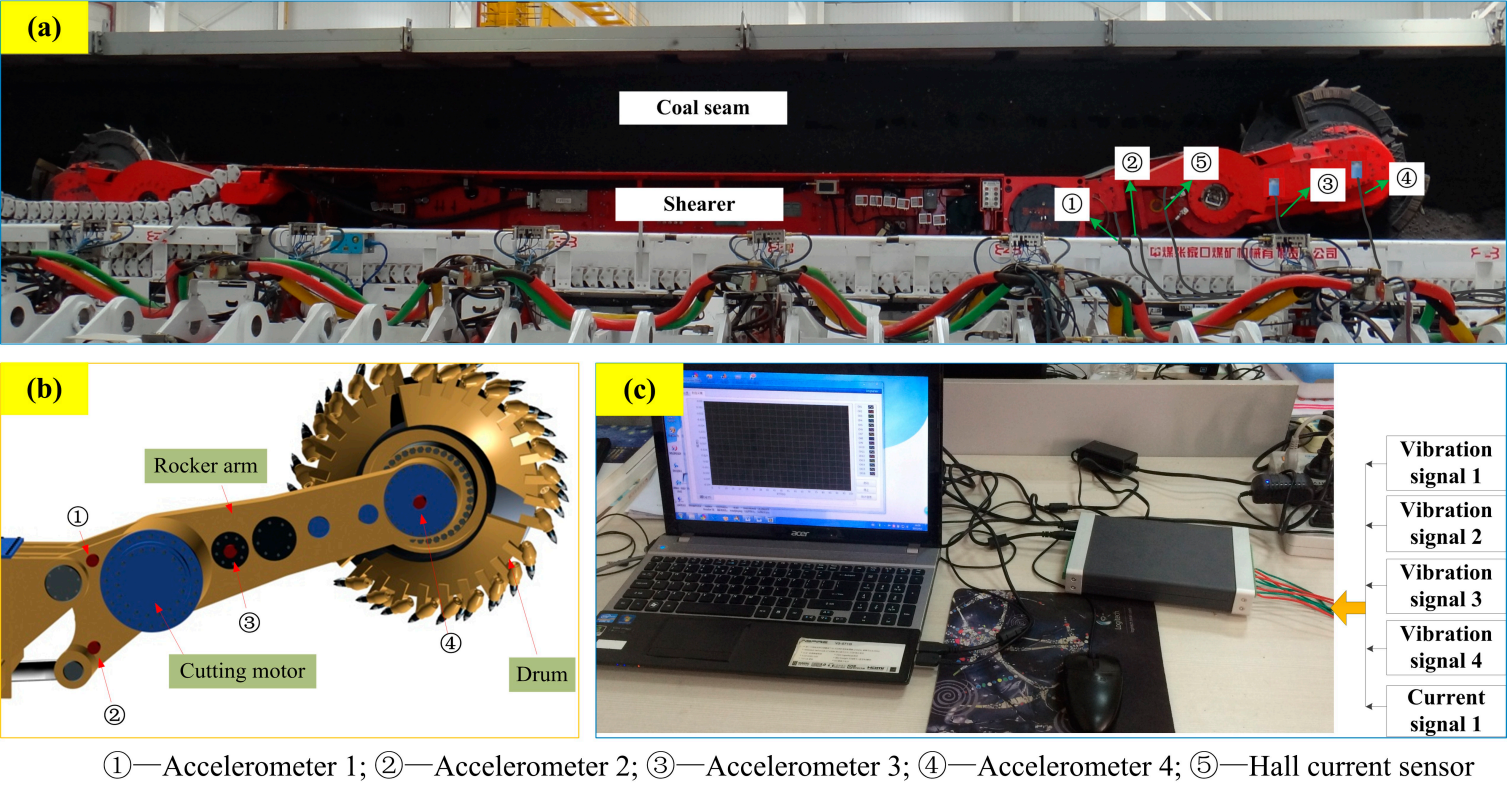
Self-designed experimental system for shearer cutting coal: (**a**) The experiment bench of shearer cutting coal; (**b**) The installation sketch of accelerometers; (**c**) Sensor signals processing device.

In order to test and verify the performance of the condition classification system, five different geological conditions of coal seams were self-designed, including three kinds of coal seams with different hardness (marked as *f* = 2, 3, 4), and the coal seam with some stratums of gangue (marked as *f* = 5, 6) as shown in Figure 2. The height of coal seam was 2 m and the length for each kind of coal seam was 10 m. When the cutting drum was unloaded, the cutting condition could be marked as *f* = 1. Then all cutting conditions of shearer drum were described in Table 1. The experiments were carried out under the self-designed test rig which was mainly composed of the shearer, accelerometers, the Hall current sensor, coal seams, data acquisition board and data processor as shown in Figure 3.

**Table 1:** Description of shearer drum cutting conditions.

Symbol	Cutting Condition
*f* = 1	Unloaded
*f* = 2	Coal seam with hardness *f* = 2
*f* = 3	Coal seam with hardness *f* = 3
*f* = 4	Coal seam with hardness *f* = 4
*f* = 5	Coal seam with gangue (diameter = 50 mm)
*f* = 6	Coal seam with gangue (diameter = 80 mm)

**Figure 4:**
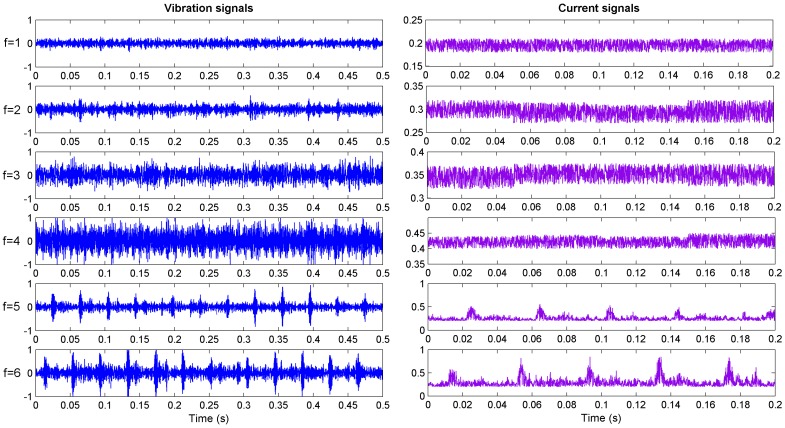
Measured signals from sensor ② and ⑤ in different conditions.

In Figure 3, the signs of “①, ②, ③ and ④” refer to four accelerometers and are used to acquire the vibration signals. The sign of “⑤” denotes the Hall current sensor and can detect the current signal of cutting motor. A multifunctional high-speed collector performs the data acquisition and the data are collected into a PC through the USB interface. The sampling frequency was set as 12 kHz and the sampling time of each sample was 0.5 s. Vibration signals of sensor ① and current signals of sensor ⑤ with different conditions were plotted in Figure 4. Finally, 600 groups of samples were obtained with 100 groups of samples for each cutting condition.

### 3.3. Feature Extraction

The feature extraction of signals is a critical initial step in any pattern recognition and fault diagnosis system. The extraction accuracy has a great influence on the final identification results. The commonly used methods for signal process are the waveform and the fast Fourier transform. However, for weak signal the features are submerged in the strong background noise and it is difficult to extract effective features by traditional feature extraction methods. Fortunately, the ensemble empirical mode decomposition (EEMD) has been proposed and it adds a certain amount Gaussian white noise in the original signal before decomposing, so as to solve the problem of frequency aliasing. This method is very appropriate for non-stationary and nonlinear signals [39,40].

The brief steps of EEMD can be summarized as follows [39]: (1) the Gaussian white noise is selected and added in the analyzed signal; (2) this new signal is decomposed by the use of EMD method and some intrinsic mode functions (IMFs) can be obtained; (3) the steps (1) and (2) are repeated to generate different IMFs through adding different Gaussian white noise; (4) the ensemble mean of IMFs are calculated and the average IMFs are regarded as the final decomposed results.

**Figure 5:**
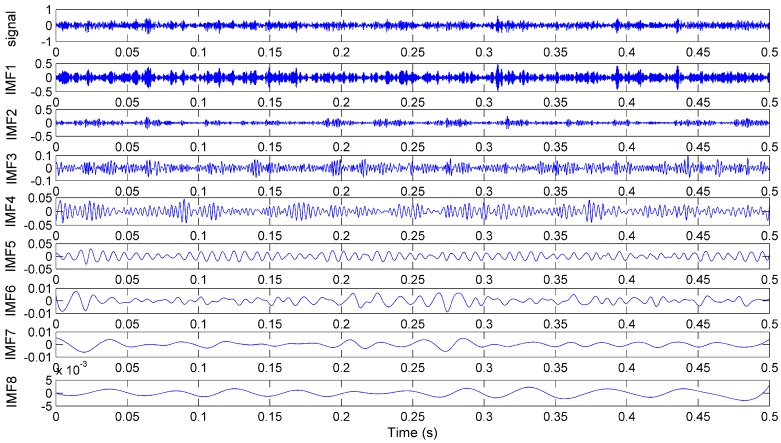
The decomposed components with EEMD for signal from sensor ① at *f* = 2.

According to the above steps, a measured signal is taken as an example to be decomposed and the decomposition results are plotted in Figure 5. It shows 8 IMFs in different frequency bands decomposed by EEMD algorithm. We can see that the original signal is very complicated and the decomposed IMFs are hard to use for state diagnosis. Therefore, features of the signals need to be extracted to obtain more useful information. As the kernel feature can comprehensively reflect a signal change and the energy of a signal will change in different frequency bands when the cutting condition changes, the two features should be extracted. Furthermore, the crest factor can reflect the signal energy concentration and kurtosis can be used to describe the degree of signal peak. In this paper, the four features including the kernel feature value, the energy value, the crest factor and kurtosis, are analyzed according to the IMFs.

#### 3.3.1. Kernel Feature Value

The signal collected by the *i*th sensor can be defined as the sequence *X_i_* = {*x*_*i*1_, *x*_*i*2_, …, *x_il_*}, where *i* = 1, 2, …, *M* and *M* is the number of sensors, *l* is the number of sampling points. Then the sequence is decomposed by EEMD to get *N* intrinsic mode components: {*IMF*_1_, *IMF*_2_, *IMF*_3_, …, *IMF_N_*}, where *IMF_k_* = {*imf*_*k*1_, *imf*_*k*2_, *imf*_*k*3_, …, *imf_kl_*}*^T^*, *k* = 1, 2, …, *N*.

The equation is defined as follows:

(10) $$e_{k}=norm\left(IMF_{k}\right)=\sqrt{\sum_{j=1}^{j=l}imf_{kj}^{2}}$$

Then, a vector can be constructed from *N* IMFs:

(11) $$NORM=\left\{e_{1},e_{2},\cdots ,e_{N}\right\}$$

A Gaussian kernel function is described as $K\left(u,v\right)=\exp\left(-\left\|u-v\right\|/2\sigma^{2}\right)$. In order to ensure the convenience of calculations, *v* is defined as a vector {0}_1×*N*_, the kernel feature value can be obtained as *kf_i_* = *K*(*NORM*, *v*) [16]. Finally, a feature sample *KF* can be obtained by calculating the *M* sensors’ signal data:

(12) $$KF=\left\{kf_{1},kf_{2},\cdots ,kf_{M}\right\}$$

After several experiments, the parameter of the Gaussian kernel function *σ* was set as 5 in this experiment. 100 groups of feature samples were extracted separately for six cutting types and the size of the sample set was 600 × 5.

#### 3.3.2. Other Three Features

IMFs decomposed by EEMD contain valid information for pattern recognition. The analysis results from EEMD energy of different signals indicate that the energy of a signal will change in different frequency bands when a condition changes. Therefore, the energy of the decomposed IMFs could be used as features for cutting condition identification [4,41]. *E_i_* is the energy of the *i*th IMF, which can be calculated as follows:

(13) $$E_{i}=\int_{-\infty }^{+\infty }{\left|IMF_{i}\right|}^{2}dt,i=1,2,\cdots ,N$$

In addition, crest factor *CF* reflects the signal energy concentration and kurtosis *Ku* is particularly sensitive to impact signal. The *CF* and *Ku* of the *i*th IMF can be calculated as follows:

(14) $$\begin{array}{l} CF_{i}=\frac{\max\left|IMF_{i}\right|}{IMF_{i}^{rms}}\\ Ku_{i}=\frac{\sum_{j=1}^{l}{\left(imf_{ij}-\bar{IMF_{i}}\right)}^{4}}{\left(l-1\right){\left(IMF_{i}^{std}\right)}^{4}} \end{array}$$

### 3.4. Classification Procedure

According to above features, different forms of sample vectors were constructed and three classifiers were preliminarily established by the use of PQN-NN. The first classifier was based on the kernel feature values of all signals, the second one was based on the energy value, crest factor and kurtosis of vibration signals, and the last one was obtained based on the features of current signal. Then the preliminarily output results of three classifiers were used to construct a set of hypotheses and the BBAs were fused by DS theory to obtain the final identification results. The specific steps can be described as follows:

(1) The discernment frame is established as *Θ* = {*A*_1_, *A*_2_, …, *A*_6_} and the propositions in *Θ* correspond to the cutting conditions.

(2) The mass of the *i*th classifier on hypothesis *A_j_* can be marked as *m_i_*(*A_j_*) and the masses are assigned only to the single hypotheses *f* = 1 to 6. This means that the choice to assign masses to singletons is equivalent to Bayes and the masses are so-called Bayesian masses, which can be calculated as follows:

(15) $$m_{i}(A_{j})=\frac{\left|C_{ij}\right|}{\sum_{j=1}^{6}\left|C_{ij}\right|}$$

where *C_ij_* is the *j*th node output of the *i*th classifier.

(3) The BBAs of three classifiers in the hypothesis are fused by the use of DS theory. In this paper, a multiple combination for the piece of evidence is applied, that is to say, the combined BBA $\hat{m}$ can be obtained by the fusion of ($m_{1}⊕m_{2}$) and *m*_3_. However, in the process of combination conflicts of *m*_1_, *m*_2_ and *m*_3_ may exist, which will be discussed in Section 3.5.4.

Then the final identification result *m_c_* of a testing sample can be computed by the following rule:

(16) $$m_{c}=\max\left\{\hat{m}(A_{j})\right\}\;\mathrm{and}\;1>A_{c}>\delta >0,\;\delta \in R$$

where *m_c_* is used to describe the confidence level of the result and *δ* is called the diagnostic reliability. This rule indicates that when the identification result *m_c_* is larger than the beforehand diagnostic reliability, we can determine that the testing sample is classified correctly.

### 3.5. Results and Discussion

#### 3.5.1. Classification of the Single Kernel Feature

The kernel feature vectors were established by feature extraction from the signals for each cutting condition. Then, 300 × 5 matrices of samples for six conditions were used as input data. As the condition classes of the feature vectors with six conditions were known, the expected output of each sample was a unit vector of dimension 6. For each cutting condition (from *f* = 1 to *f* = 6), the corresponding outputs could be defined as (1, 0, 0, 0, 0, 0), (0, 1, 0, 0, 0, 0), (0, 0, 1, 0, 0, 0), (0, 0, 0, 1, 0, 0), (0, 0, 0, 0, 1, 0), and (0, 0, 0, 0, 0, 1), respectively. The parameters of the advanced network were listed as follows: the nodes of input layer *n*_1_ = 5; the nodes of hidden layer *n*_2_ = 2*n*_1_ + 1 = 11; the nodes of output layer *n*_3_ = 6; *K*_max_ = 1000; *ε*_1_ = 0.5, *ε*_2_= 1; *ρ*_1_= 0.0001, *ρ*_2_= 0.9; *ε* = 0.0001. Fifty feature vectors of each cutting condition, totaling 300 samples, were used to train the PQN-NN. The testing set, composed of the surplus 50 feature vectors, was only used for testing the generalization of the neural network after it was trained. At that point, we could obtain the first classifier, marked as *C*_1_. The mean of 50 testing results for each cutting condition was shown in Table 2. We adopt the maximum value of the six node outputs as the identified results. The classification accuracy can be calculated as the number of classified samples/the number of testing samples ×100%. Thus the accuracies for the six cutting types are 88%, 84%, 86%, 84%, 88% and 88%, respectively. The diagnostic result is accurate. It demonstrates that the developed PQN neural network only using the single feature has good performance in identifying the cutting conditions.

**Table 2:** Testing results of PQN neural network based on single kernel feature.

Test Pattern	Mean of Network Outputs					
	*f* = 1	*f* = 2	*f* = 3	*f* = 4	*f* = 5	*f* = 6
*f* = 1	0.7619	0.1523	0.0881	0.0687	0.0297	0.0349
*f* = 2	0.0981	0.7216	0.1075	0.1985	0.1524	0.1158
*f* = 3	0.0846	0.1575	0.6846	0.2075	0.1106	0.0954
*f* = 4	0.0246	0.0978	0.1256	0.8254	0.1052	0.0465
*f* = 5	0.0159	0.0275	0.1278	0.0985	0.8985	0.2045
*f* = 6	0.0267	0.0598	0.0684	0.0468	0.2241	0.8551

In order to test the effectiveness and superiority of PQN-NN, other models were selected to compare with the proposed model. Considering the extensive application of support vector machine (SVM) and BP neural network (BP-NN), the BP-NN and SVM models are built by the features to identify the shearer cutting condition. The compared results were shown in Table 3. It indicates that the advanced network achieved the best diagnostic performance. Besides, the average identification accuracy of BP-NN, SVM and PQN-NN is 82.67%, 84.67%, 86.33%, respectively. The comparison results show that the PQN neural network outperforms the other two common methods in identifying different cutting conditions of a shearer.

**Table 3:** Testing accuracy of BP-NN, SVM and PQN-NN.

Test pattern	Samples	BP-NN	SVM	PQN-NN
*f* = 1	50	82%	84%	88%
*f* = 2	50	84%	82%	84%
*f* = 3	50	80%	84%	86%
*f* = 4	50	82%	84%	84%
*f* = 5	50	86%	88%	88%
*f* = 6	50	82%	86%	88%

#### 3.5.2. Classification of the Vibration Signals

The second classifier (*C*_2_) could be established based on the features of energy, crest factor and kurtosis extracted from the vibration signals. According to the analysis in Section 3.3, the strongest IMFs of each vibration signals were used to establish the training samples of PQN-NN. An input vector of network could be selected as follows. In the first scheme, the input vector $P_{1}=\left[E_{1\max},\cdots ,E_{4\max},CF_{1\max},\cdots ,CF_{4\max},Ku_{1\max},\cdots ,Ku_{4\max}\right]$ contained a total of 12 elements, where $E_{i\max}$, $CF_{i\max}$ and $Ku_{i\max}$ represented the maximum energy, crest factor and kurtosis of IMFs for the *i*th vibration signal. In the second scheme, the two largest feature values of IMFs were used to construct the input vector ***P***_2_, which was composed of 24 elements. Therefore, the nodes of input layer *n*_1_ = 12 and 24, the nodes of hidden layer *n*_2_ = 2*n*_1_ + 1 and the nodes of output layer *n*_3_ = 6. Other parameters were consistent with previous network model. The training set was composed of 300 feature vectors with 50 samples for each cutting condition and the remaining 300 feature vectors were used to test the trained networks. In order to verify the superiority of the schemes, four signals were separately utilized to identify the cutting conditions. The input vector was selected as $\left[E_{i\max1},E_{i\max2},CF_{i\max1},CF_{i\max2},Ku_{i\max1},Ku_{i\max2}\right]$. The compared identification results of each cutting condition were shown in Table 4.

**Table 4:** Classification accuracy of classifiers based on vibration signals.

Test Pattern	Scheme 1	Scheme 2	Sensor ①	Sensor ②	Sensor ③	Sensor ④
*f* = 1	84%	88%	78%	80%	78%	82%
*f* = 2	84%	86%	82%	82%	78%	84%
*f* = 3	82%	82%	80%	82%	76%	80%
*f* = 4	86%	86%	86%	86%	80%	86%
*f* = 5	86%	88%	84%	84%	82%	84%
*f* = 6	84%	82%	80%	76%	74%	80%
Average	84.33%	85.33%	81.67%	81.67%	78%	82.67%
Classification time	13.56 s	25.15 s	7.78 s	7.93 s	7.56 s	7.24 s

Seen from Table 4, the classifiers based on schemes 1 and 2 can obtain higher classification accuracy than the classifiers based on a single vibration signal, although the classification time is a little longer than others. Compared with scheme 2, the classifier based on scheme 1 combines the most significant information of four signals and performs with almost the same recognition rate, but the classification time is reduced by about 46 percent. In conclusion, the classifier based on scheme 1 represents good comprehensive properties and can be utilized to construct the second classifier *C*_2_.

#### 3.5.3. Classification of the Current Signal

**Figure 6:**
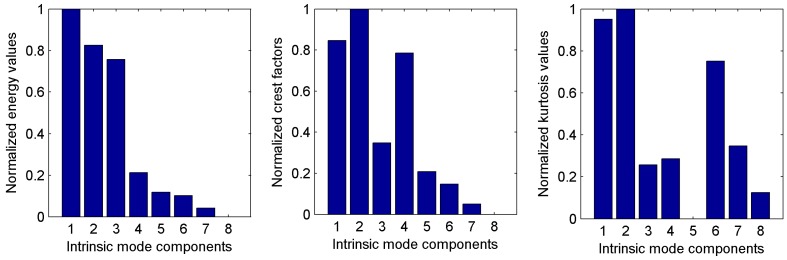
Normalized features of IMFs decomposed from current signal.

Because the single current signal was only collected from sensor ⑤, the third classifier (*C*_3_) could be easily constructed by the PQN-NN model. The current signal was decomposed by EEMD, then the energy, crest factor and kurtosis of each IMF were calculated. The [0, 1] normalization for the feature values could be achieved through Equation (17) and the corresponding results were plotted in Figure 6.

(17) $${\hat{x}}_{ij}=\frac{x_{ij}-x_{i\min}}{x_{i\max}-x_{i\min}}$$

where *i* = 1, 2, 3 and denotes the energy, crest factor and kurtosis, respectively; *j* = 1, 2, …, 8 and denotes the *j*th IMF; *x_ij_* denotes the *i*th feature value of the *j*th IMF; *x*_*i*min_ and *x*_*i*max_ denote the minimum and maximum values of the *i*th feature among the 8 IMFs.

Seen from Figure 6, the energy mainly distributes in the first three IMFs. The crest factors are distinctly superior in the first, second and fourth IMFs. The kurtosis values of IMF1, IMF2 and IMF6 are obviously higher than others. Therefore, the current signal could be represented by an eigenvector composed of 3 energy values, 3 crest factors and 3 kurtosis values. Thus, an input vector of the network was selected as $Q=\left[E_{1},E_{2},E_{3},CF_{1},CF_{2},CF_{4},Ku_{1},Ku_{2},Ku_{6}\right]$. The nodes of input layer *n*_1_ = 9, the nodes of hidden layer *n*_2_ = 2*n*_1_ + 1 = 19 and the nodes of output layer *n*_3_ = 6. Other parameters were consistent with previous network model. The training set and testing set both were composed of 50 feature vectors for each cutting condition. The final diagnosis results were provided in Table 5, where the classification error was the relative error between the output results and desired values of classified samples and could be calculated as |output results − desired values|/desired values × 100%. The classification error based on current signal can reach 15.54%, which indicates that the outputs of PQN-NN have obvious difference from ideal outputs and the credibility of the network outputs is lower, although the classified results are correct. In addition, the mean classification accuracy can fall to below 80%, only 79.67%. The simulation results are not faultless and cannot be completely accepted.

**Table 5:** Classification accuracy of classifier based on current signal.

Test Pattern	*f* = 1	*f* = 2	*f* = 3	*f* = 4	*f* = 5	*f* = 6	Average
Classification error	13.45%	18.75%	21.74%	12.84%	11.66%	14.78%	15.54%
Classification accuracy	82%	78%	76%	82%	78%	82%	79.67%

#### 3.5.4. Combination of Three Classifiers

In this subsection, in order to improve the identification performance of single classifiers, the DS theory is used to fuse the outputs of three classifiers. Firstly, the three classifiers (*C*_1_, *C*_2_ and *C*_3_) were trained separately to obtain the output results based on different feature vectors. Then, the outputs of the classifiers were processed through Equation (15) to generate the BBA *m* (so-called Bayesian mass) and sensor fusion mechanism was realized through the combination of several belief functions based on Dempster’s rule.

According to Section 3.4, the frame of discernment *Θ* = {*A*_1_, *A*_2_, …, *A*_6_} and *A_i_* is corresponding to each cutting condition. For each classifier, the BBA *m* of *A_i_* could be calculated through Equation (15). For example, Table 6 presented the combination process of the outputs of three classifiers for test pattern *f* = 2 by the DS theory. Although the test pattern *f* = 2 has lower BBA *m* in classifier *C*_3_, the pattern has higher BBA *m* than other classes in classifier *C*_1_ and *C*_2_. After the combination process through DS theory, the combined BBA *m* of pattern *f* = 2 is 0.9956. The obtained results are perfect compared with individual classifiers primarily because the use of BBA can avoid the contradictory mass assignment problem.

**Table 6:** Combination process of the outputs of three classifiers about one test sample of *f* = 2.

Classifier	*f* = 1	*f* = 2	*f* = 3	*f* = 4	*f* = 5	*f* = 6
*C*_1_	0.0895	0.7503	0.0754	0.0551	0.0119	0.0178
*C*_2_	0.0774	0.8207	0.0524	0.0164	0.0115	0.0216
*C*_3_	0.1544	0.5849	0.1064	0.0625	0.0347	0.0571
Combined BBA $\hat{m}$	0.002956	0.9956	0.001162	0.001560	1.31e−5	6.06e−5

**Figure 7:**
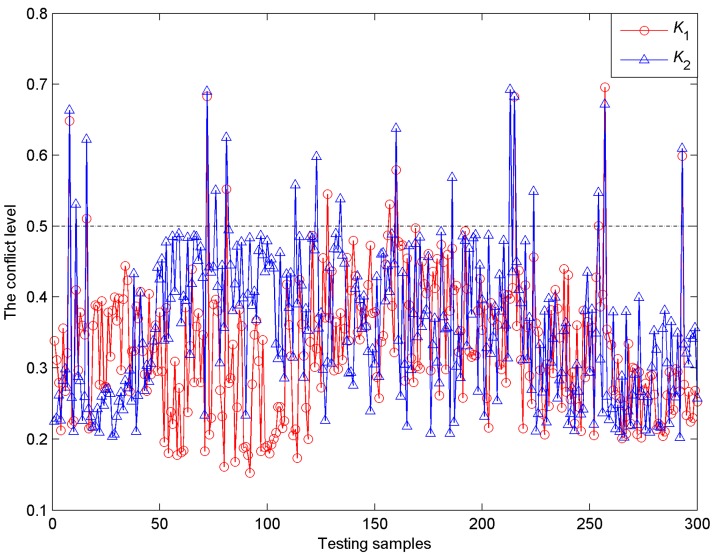
The conflict levels obtained for 300 testing samples in the combination process.

However, in the process of combination the conflicts of *m*_1_, *m*_2_ and *m*_3_ may exist and it is necessary to discuss the fact that the three classifiers may have contradictory outputs. In DS theory, the degree of conflict *K* is utilized to describe the conflict level. In this case, *K*_1_ is used as the conflict level in the fusion process of *m*_1_ and *m*_2_, and *K*_2_ is used as the conflict level in the fusion process of ($m_{1}⊕m_{2}$) and *m*_3_. The two conflict levels obtained for all testing samples are illustrated in Figure 7. Seen from this figure, the conflict levels of only 19 testing samples are above or equal to 0.5 and most conflict values are less than 0.5. The results indicate that some conflicts may only exist in the combination of the 19 samples and no conflict or lower conflict exists in the combination of the remaining 281 samples. The conflict may have some influences on the final classification results, which will be discussed in the following parts. Therefore, in this experiment, using conventional DS theory to combine the BBAs is reasonable and the identification results are obviously improved.

**Figure 8:**
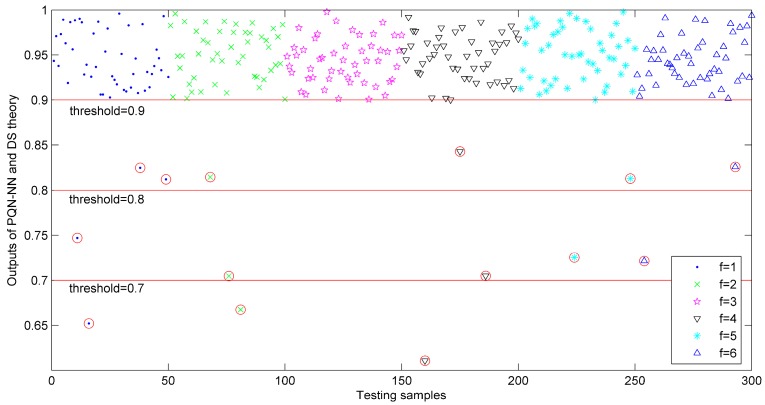
Output results based on PQN-NN and DS theory for 300 testing samples.

The detection results using PQN-NN and DS theory of each cutting condition were shown in Figure 8, where the diagnostic threshold was used to determine the credibility of outputs. If the fused results of testing samples were smaller than this threshold, these samples were be rejected and marked as wrongly classified samples. In this simulation, the diagnostic threshold *δ* was set as 0.7, 0.8 and 0.9, respectively. Seen from Figure 8, most fused results are effective and the useless testing samples for different thresholds (0.7, 0.8 and 0.9) are circled in red.

Concerning the threshold *δ* = 0.8, the classification results of the testing samples based on proposed method is shown in Figure 8. As shown in this figure, only four testing samples are misclassified and circled in red. The classification accuracies (including the useless samples) for six cutting conditions are 94%, 94%, 100%, 96%, 96% and 96%, respectively. Overall average classification accuracy is 96%. The same method can be used to analyze the classification accuracies for other diagnostic thresholds and the results are listed in Table 7.

**Figure 9:**
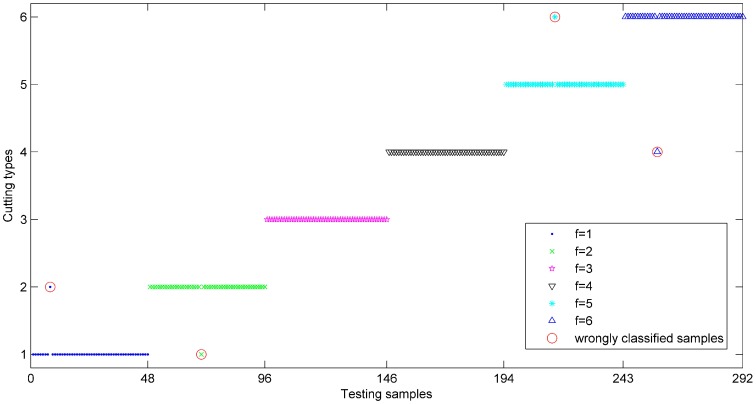
Classification results based on combined classifier at *δ* = 0.8.

In order to compare the combined classification performance with single classifiers, the outputs of classifiers *C*_1_, *C*_2_ and *C*_3_ were processed through Equations (15) and (16) to compute the identification accuracies. Finally, the classification results based on different classifiers were compared and listed in Table 7. We can see that the combined diagnostic accuracies for six cutting conditions are 96%, 98%, 100%, 98%, 100% and 98%, respectively and the average classification accuracy can reach 98.33% at *δ* = 0.7, which are superior to the individual classifiers (88%, 87% and 86%). When *δ* = 0.9, the combined accuracies for six cutting conditions are 90%, 92%, 98%, 94%, 96% and 94%, respectively and the average accuracy can reach 94%, which are obviously higher than those of individual classifiers (79%, 78.33% and 75.67%). With the increase of threshold value, the diagnostic accuracies of all classifiers represent a downward trend and the combined results can remain 94% at *δ* = 0.9, which is very accredited. Comparing the identification results with Figure 7, most misclassified samples appear among the testing samples with unsatisfactory conflicts in the combination process of DS theory and we can think that the main reason for the misclassified samples may be the conflicts. Furthermore, the combined classifier would generate a minimum number of three useless samples other than single classifiers (12, 14 and 15) at *δ* = 0.7. When *δ* = 0.8 and 0.9, the numbers of useless samples for the combined classifier are 8 and 14, which are obviously smaller than other single classifiers (20, 18, 23 for *δ* = 0.8 and 28, 33, 32 for *δ* = 0.9). This comparison indicates that the outputs of the proposed method have higher credibility than others. In general, the compared results manifest that the combined identification method based on PQN-NN and DS theory is feasible and can be applied in the identification of shearer cutting conditions. 

**Table 7:** Comparison of classification results based on different classifiers with different *δ*.

Diagnostic Threshold	Test Pattern	*C*_1_	*C*_2_	*C*_3_	Combined Results
*δ* = 0.7	Useless samples	12	14	15	3
	*f* = 1	88%	86%	84%	96%
	*f* = 2	86%	84%	84%	98%
	*f* = 3	86%	88%	86%	100%
	*f* = 4	90%	86%	88%	98%
	*f* = 5	88%	90%	88%	100%
	*f* = 6	90%	88%	86%	98%
	Average	88%	87%	86%	98.33%
*δ* = 0.8	Useless samples	20	18	23	8
	*f* = 1	84%	82%	80%	94%
	*f* = 2	82%	82%	82%	94%
	*f* = 3	86%	86%	84%	100%
	*f* = 4	84%	88%	80%	96%
	*f* = 5	80%	82%	86%	96%
	*f* = 6	82%	80%	82%	96%
	Average	83%	83.33%	82.33	96%
*δ* = 0.9	Useless samples	28	33	32	14
	*f* = 1	74%	74%	72%	90%
	*f* = 2	76%	72%	70%	92%
	*f* = 3	80%	78%	76%	98%
	*f* = 4	82%	80%	76%	94%
	*f* = 5	82%	84%	82%	96%
	*f* = 6	80%	82%	80%	94%
	Average	79%	78.33%	75.67%	94%

#### 3.5.5. Comparison with Other Methods

In this section, the classifier based on single BP-NN and PQN-NN was established in this paper to test and verify the classification performance of the proposed method. The configurations of the simulation environment for the algorithms were uniform and in common with above experiments. According to the above analysis, a sample of BP-NN or PQN-NN could be composed of the kernel feature *KF_i_* and the maximum energy *E*_*i*max_, crest factor *CF*_*i*max_, kurtosis *Ku*_*i*max_ of IMFs for the *i*th signal. Therefore, the nodes of input layer *n* = 20 and the nodes of output layer *n*_3_ = 6. Other parameters were consistent with previous network model. In order to avoid the random error, the training set of input was presented to the networks 10 times and the average values were calculated. Finally, the comparison of classification time and accuracy based on three algorithms was shown in Table 8.

**Table 8:** Comparison of classification results through BP-NN, PQN-NN and the proposed classifier.

Diagnostic Threshold	Classifier Types	Classification Time /s	Classification Accuracy
*δ* = 0.7	BP-NN	19.7845	85.87%
	PQN-NN	25.6456	89.76%
	Proposed classifier	23.1245	98.27%
*δ* = 0.8	BP-NN	19.7845	81.63%
	PQN-NN	25.6456	86.13%
	Proposed classifier	23.1245	95.87%
*δ* = 0.9	BP-NN	19.7845	75.37%
	PQN-NN	25.6456	82.13%
	Proposed classifier	23.1245	93.93%

From the table, it can be observed that the proposed method has a better classification capability and performance than the competing methods. With the benefits of DS theory in uncertain fields, the proposed classifier can obtain higher classification accuracy than single BP-NN and PQN-NN classifiers in different diagnostic thresholds, although the classification time is not excellent. In general, the proposed classifier is proved feasible and superior to the competing algorithms.

In order to verify the performance gain from using a parallel computing in NNs, the convergence condition *ε* was set as 0.01, 0.001 and 0.0001, respectively, and the maximum iterations *K*_max_ was set as 1000. The convergence condition was the first termination criterion for iteration and the maximum iterations was the second termination criterion for iteration. We adopted the classification results obtained by BP-NN, PQN-NN and proposed classifier at diagnostic threshold *δ* = 0.8 to compare and analysis the convergence performance. The results obtained by different algorithms were shown in Table 9.

**Table 9:** Results obtained by different algorithms at *δ* = 0.8.

Convergence Condition	Iterations			Classification Time/s			Classification Accuracy		
	BP-NN	PQN-NN	Proposed Classifier	BP-NN	PQN-NN	Proposed Classifier	BP-NN	PQN-NN	Proposed Classifier
0.01	428	101	86	8.67	3.67	2.46	72.44%	78.57%	86.27%
0.001	747	315	274	16.84	10.87	9.25	76.18%	82.66%	90.15%
0.0001	1000	724	648	19.78	25.65	23.12	81.63%	86.13%	95.87%

Seen from Table 9, the classification accuracy is distinctly higher than those of other classifiers with different convergence conditions. When *ε* = 0.0001, the iterations of BP-NN reach the maximum value, while the iteration of PQN-NN and the proposed algorithm are only 724 and 648, which causes their classification times (25.65 s and 23.12 s) to be a little longer than that of BP-NN. With the increase of *ε*, the convergence speeds of NNs with a parallel computing algorithm are surprisingly faster than that of NNs with a BP algorithm. In detail, the iterations and classification times of PQN-NN and proposed algorithm are significantly lower than those of BP-NN, the reason for which is that two parallel search directions are used in PQN-NN and the proposed algorithm to increase the convergence speed.

## 4. Industrial Application

**Figure 10:**
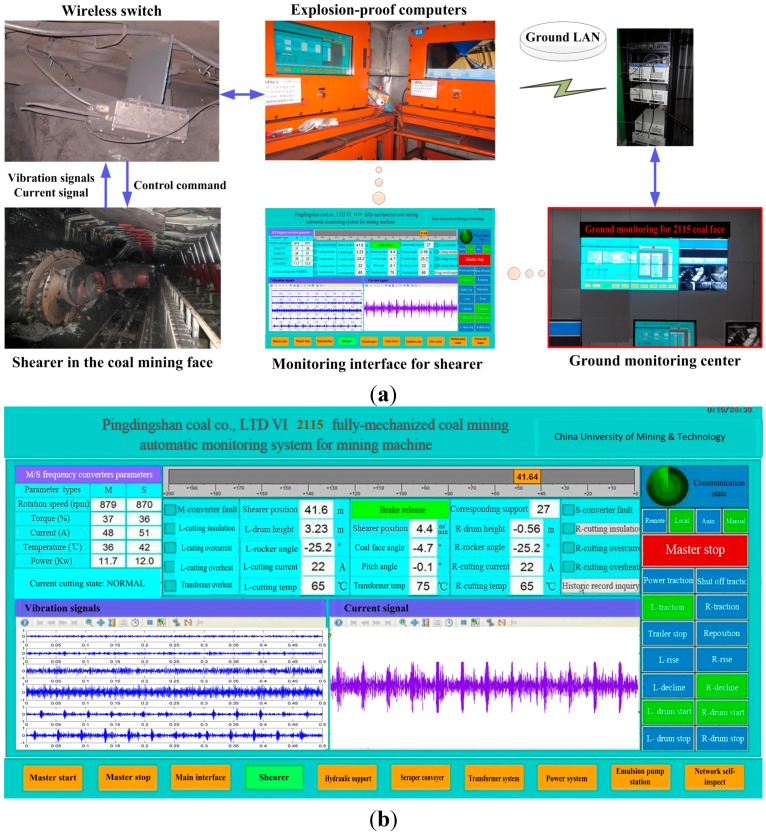
Industrial application example of proposed method: (**a**) The system in coal mining face based on proposed method; (**b**) The monitoring interface for shearer.

In this section, an online system based on proposed approach had been developed and applied in the field of actual fully mechanized coal mining face as shown in Figure 10.

**Figure 11:**
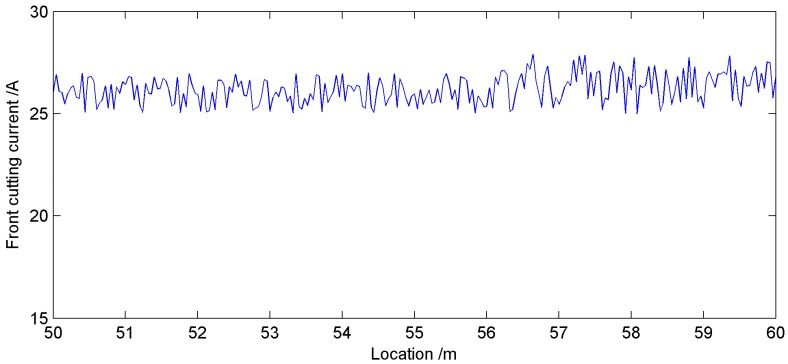
Change curve of shearer left cutting current from 50 m to 60 m.

In the industrial experiment, the explosion-proof accelerometers and current sensor were installed on the shearer arm shell. The collected signals were transmitted into an explosion-proof computer through some sireless switches on the coal mining face. The explosion-proof computer could process the signals to identify the cutting condition of shearer and display them on the monitoring interface. According to identification results based on the proposed system, the shearer control system could send proper control commands to keep reasonable cutting state. When the shearer was cutting the coal seam from 50 m to 60 m, the change curve of front cutting current was plotted in Figure 11. The curve was smooth and the sharply mutations were avoided, which proved the availability and reliability of the online system based on proposed method.

## 5. Discussion and Conclusions

### 5.1. Conclusion

The main contribution of this paper is that a methodology based on parallel quasi-Newton neural network (PQN-NN) and Demspter-Shafer (DS) theory for the identification of shearer cutting condition is presented. In this method, PQN-NN is used as a classifier constructed by the features to identify the cutting condition of shearer and the extracted features from the measured signals presenting the state of the shearer are used for inputs to the classifiers. The classification results of six cutting conditions are assessed quantitatively and combined to achieve data fusion by DS’s combination rule. The experimental results demonstrate that the proposed method performed higher classification accuracy and better generalization ability than the classifiers based on single neural network methods. Furthermore, the industrial application indicates that the system based on the proposed method can provide stable and reliable references for the automatic control of a shearer. It is an effective method for state detection of heavy-duty machinery working in noisy and complicated environments.

### 5.2. Discussion 

The feature extraction step of the proposed method is accomplished by extracting features from vibration signals and cutting current signal based on EEMD, because it is easy for non-expert to attain without much experience. The condition identification results may be further improved if advanced feature extraction methods are adopted.

Compared with other methods, the proposed method of parallel quasi-Newton neural network (PQN-NN) with Dempster-Shafer (DS) theory improves the cutting condition diagnosis result and diagnosis time, and acquires desirable accuracy, which testifies that good diagnosis performance not only depends on extracting proper features but also depends on combining recognition results from multiple sensors. In future works, the authors will consider the great advantage of DS theory and assign the masses to subsets of hypotheses. This can avoid the conflict in the combination process and the final combination results based on DS theory can be further improved.

The proposed method has proved to be effective in intelligent cutting pattern identification of shearers by the experiments and industrial application in the actual fully mechanized coal face. The proposed method can also be applied in the state detection of other machinery to achieve success.
